# A “Do No Harm” Novel Safety Checklist and Research Approach to Determine Whether to Launch an Artificial Intelligence–Based Medical Technology: Introducing the Biological-Psychological, Economic, and Social (BPES) Framework

**DOI:** 10.2196/43386

**Published:** 2023-04-05

**Authors:** Waqas Ullah Khan, Emily Seto

**Affiliations:** 1 Health Informatics Institute of Health Policy, Management and Evaluation University of Toronto Toronto, ON Canada

**Keywords:** artificial intelligence, AI, safety checklist, Do No Harm, biological-psychological factors, economic factors, social factors, AI medical hardware devices, AI medical mobile apps, AI medical software programs

## Abstract

Given the impact artificial intelligence (AI)–based medical technologies (hardware devices, software programs, and mobile apps) can have on society, debates regarding the principles behind their development and deployment are emerging. Using the biopsychosocial model applied in psychiatry and other fields of medicine as our foundation, we propose a novel 3-step framework to guide industry developers of AI-based medical tools as well as health care regulatory agencies on how to decide if a product should be launched—a “Go or No-Go” approach. More specifically, our novel framework places stakeholders’ (patients, health care professionals, industry, and government institutions) safety at its core by asking developers to demonstrate the biological-psychological (impact on physical and mental health), economic, and social value of their AI tool before it is launched. We also introduce a novel cost-effective, time-sensitive, and safety-oriented mixed quantitative and qualitative clinical phased trial approach to help industry and government health care regulatory agencies test and deliberate on whether to launch these AI-based medical technologies. To our knowledge, our biological-psychological, economic, and social (BPES) framework and mixed method phased trial approach are the first to place the Hippocratic Oath of “Do No Harm” at the center of developers’, implementers’, regulators’, and users’ mindsets when determining whether an AI-based medical technology is safe to launch. Moreover, as the welfare of AI users and developers becomes a greater concern, our framework’s novel safety feature will allow it to complement existing and future AI reporting guidelines.

## Introduction

Artificial intelligence (AI) can be described as the theory and development of computer systems able to perform tasks that normally require human decision-making [1]. In medicine, the idea that clinical decisions can be made almost instantly using health care data spanning multidisciplinary domains seemed impossible 3 decades ago. However, with AI algorithm-based medical technologies, diagnoses and care plans that use a large repository of health care data are nearing real-time application for personalized medicine [2].

The use of these tools promises to significantly improve health care delivery, reduce wait times and costs, and expand access to care worldwide [2]. Nevertheless, it would be naïve to think that AI technologies are infallible. For instance, they can be developed for unethical reasons, such as Volkswagen’s AI algorithm used to pass emission tests with environmentally unfavorable vehicles. Private-sector AI medical technology companies might also be subject to similar temptations, especially when reimbursement rates are based on metrics that do not always reflect better care [3].

In light of the impact AI-based medical technologies can have on society, debates regarding the principles behind their development and deployment are emerging. National and international organizations have responded to these concerns by creating ad-hoc expert committees to develop policies and guidelines [1]. This includes the European Commission’s High-Level Expert Group on Artificial Intelligence, the AI in society expert group of the Organization for Economic Co-operation and Development (OECD), the US Food and Drug Administration (FDA), Health Canada, and the United Kingdom’s Medicines and Healthcare Products Regulatory Agency’s (MHRA) joint development of “Good Machine Learning Practice” principles [1,4].

Private sector corporations have also created policies regarding AI technologies. For instance, in 2018, Google and SAP publicly disclosed their AI guidelines and principles. Recommendations for AI technology guidelines and principles have also been made by nonprofit organizations such as Amnesty International [1]. Nevertheless, most AI policies, guidelines, and frameworks are often designed for a select group of stakeholders, use language unfamiliar to health care professionals and patients, often provide guidance only on the implementation, adoption, evaluation, and regulatory aspects of AI medical tools, and overlook the Hippocratic Oath of “Do No Harm” in deciding whether it is safe to launch a product.

**Figure 1:**
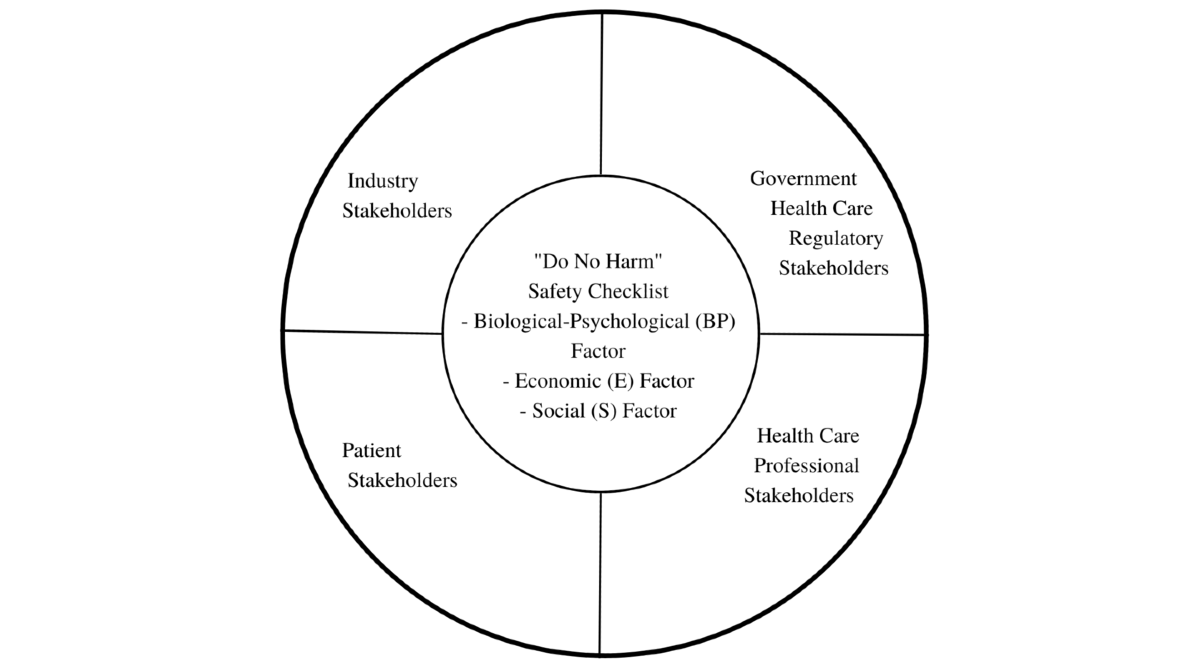
The “Do No Harm” biological-psychological, economic, and social safety checklist framework for assessing the launch of artificial intelligence–based medical technologies.

The biopsychosocial model was developed over 40 years ago to help understand the etiology of medical conditions and formulate care plans from a multidisciplinary perspective. This is achieved by examining the relationship between the pathophysiology (biological), psychological, and social factors involved in a medical condition [5]. Using the biological-psychological, economic, and social (BPES) model as our foundation, we propose a 3-step framework to guide developers of AI-based medical technologies (hardware devices, software programs, and mobile apps) on how to decide if they should launch their tool—a “Go or No-Go” approach. More specifically, our framework places stakeholders’ (patients, health care professionals, industry, and government institutions) safety at its core by asking developers to demonstrate the BPES value of their AI tool before it is launched (Table 1 and Figure 1).

**Table 1:** The biological-psychological, economic, and social framework safety checklist.

Stakeholders	Biological-psychological perspective—stakeholder safety research evidence provided (yes or no with supporting evidence)	Economic perspective—stakeholder safety research evidence provided (yes or no with supporting evidence)	Social perspective—stakeholder safety research evidence provided (yes or no with supporting evidence)
Patients			
Health care professionals			
Industry			
Government—public health agency			

The novel safety feature of the BPES framework also separates it from other AI medical technology models by addressing the Collingridge dilemma. This occurs when policy making regarding a new technology is delayed as the impact on society cannot be predicted until it is extensively developed and used. However, once the technology is engrained in society and its harmful effects become evident, it might be too late to act [6]. Moreover, as the welfare of AI users and developers becomes a greater concern, our framework’s novel safety feature will allow it to complement existing and future AI reporting guidelines such as “DECIDE-AI” (Developmental and Exploratory Clinical Investigations of Decision support systems driven by Artificial Intelligence) [7]. This guideline was recently launched and developed for early stage clinical evaluation of decision support systems that are driven by AI. The DECIDE-AI guideline checklist recommends the user apply a framework (Human Factors) along with a method to identify and discuss the safety as well as errors of an AI tool [7]. The BPES framework can facilitate this by providing a context of safety and errors to consider (BPES) when clinically evaluating an AI tool during its early stage.

## Biological-Psychological Factor—Does the AI Medical Technology Cause Physical-Mental Health Benefit or Harm to the Stakeholders Involved?

### Patient Perspective

When determining if an AI medical technology should be launched, an assessment of whether it improves a clinical outcome (ie, medical diagnosis, prognosis, and care plan; biological-psychological factor) without increasing the risk of harm to the patient should be considered. For example, a novel deep learning architecture has been developed that uses a heterogeneous set of 3D optical coherence tomography scans to diagnose and refer retinal disease [8]. More specifically, this AI algorithm classifies the findings from scans into different pathologies while providing clinicians with care plan recommendations (urgent, semiurgent, routine referral, or observation). Remarkably, this AI medical tool has reached or exceeded expert decision-making on a range of vision-threatening retinal diseases. Moreover, it reduces the risk of harm to patients as its aggregated number of wrong referral decisions is lower than human experts (retina specialists and optometrists) [8].

### Health Care Professional Perspective

An AI medical technology should also provide health care professionals with an accurate diagnosis, prognosis, and effective care plan without increasing their risk of harm or work-related burden. For example, an individual presenting to the emergency department with sudden onset breathlessness can receive numerous differential diagnoses and management of care plans. Nevertheless, an effective AI medical tool should assist clinicians in making an accurate diagnosis, prognosis, and care plan based on the data obtained from the patient’s history, examination, and investigations. Moreover, it should not create confusion, consume more resources, or lengthen the duration required to establish a diagnosis, prognosis, and care plan.

### Industry and Government Perspectives

From industry and government perspectives, AI technologies should reduce medical risks to the population and improve public health measures. For instance, one AI tool passively listened to individuals speak on their cellphone, recognized speech patterns associated with “thought disorder,” and predicted the onset of a psychotic episode in high-risk youth. With 100% accuracy, this AI algorithm identified several individuals who later developed psychosis and outperformed identification from clinical interviews [9]. In addition to predicting disease, medical technologies that use AI should provide rapid and accurate information to public health organizations. For example, an AI medical tool mined data from over 5 million Twitter posts and predicted a flu outbreak with an accuracy of 0.89. This AI technology used natural language processing and outperformed the current advanced public health methods used [10].

### Biological-Psychological “No-Go” Case

Unfortunately, from a biological “Do No Harm” perspective, not all developers of AI medical technologies demonstrate stakeholder safety before launching their products. For instance, wearable devices using AI tools such as the “Apple Watch” were marketed to detect atrial fibrillation and provide guidance to the user. Several studies have since evaluated this claim, which the company justified by stating 700,000 Americans with atrial fibrillation remain undiagnosed at a cost of US $3.2 billion in potentially avoidable arrhythmia-related strokes [11,12]. However, this wearable device was found to capture false-positive results that increased anxiety among participants as well as the number of misdiagnoses of atrial fibrillation among young adults with a low pretest probability of having an arrhythmia [12-14].

In this example, the product not only created more harm to the patient by providing a misdiagnosis and increasing their anxiety but also increased the workload on health care professionals who had to manage the influx of patients presenting with a query of atrial fibrillation. The industry was also harmed as similar smartwatch devices had their diagnostic credibility questioned. Finally, public health officials were harmed due to an increased burden on their resources to assess the medical claims made by these technologies and inform the population of any concerns when using them. Had there been a “Do No Harm” stakeholder checklist that required Apple to demonstrate its smartwatch’s safety from a biological-psychological perspective, its launch may have been delayed in favor of further product testing.

## Economic Factor—Does the AI Medical Technology Cause Economic Benefit or Harm to the Stakeholders Involved?

The importance of having a value-based health care system is fundamental given its share in the overall economy of many developed countries. For instance, approximately 18% of the total gross domestic product of the United States, roughly US $3.5 trillion, is used on health care expenditures [15]. Similarly in Europe, Germany spends approximately 11.5% of its overall gross domestic product (US $0.4 trillion) on health care costs [15]. For this reason, another factor to consider when deciding if an AI medical technology should be launched is whether its economic benefits outweigh the costs incurred by the stakeholders involved.

### Patient Perspective

From a patient perspective, an economic “Do No Harm” safety checklist would favor AI medical technologies that reduce their financial burden while providing improved or equally effective care when compared with the standard used. This is seen in the FDA’s recently approved autonomous AI diagnostic system to detect diabetic retinopathy (DR) in adults [16]. Using this tool, a minimally trained operator supported by AI takes retinal images using a nonmydriatic fundus camera. These images are then assessed in real time at the point of care for the presence of DR [16]. In 2020, this autonomous AI tool became a part of the American Diabetes Association’s standard of care for DR screening. A subsequent study examined its cost-effectiveness in pediatric patients and found it more efficacious in diagnosing DR and provided cost-savings (reduced out-of-pocket payments) than the standard referral-based approach using ophthalmologists or optometrists for screening examinations [16].

### Health Care Professional Perspective

Deciding whether an AI medical technology is launched should also be based on weighing the economic benefits and harms to health care professionals. One example of this is to provide financial incentives to physicians who reduce unnecessary spending and wasteful care. This can be done using an AI tool that identifies practitioners who are more likely to make high-value and cost-effective decisions [17]. In a recent study, the variation among physicians in delivering low-value health care services and their predicted characteristics was investigated [18]. Using Medicare claims of 3,159,834 beneficiaries served by 41,773 primary care physicians, the rates of low-value services were 60% higher for family physicians at the 90th percentile of their provider organization compared with those in the 10th percentile. Moreover, only 1.4% of physician variation was explained by observable characteristics. Since visible characteristics could not explain the variation in physicians providing low-value care, an AI tool that measures this might be used to identify and incentivize cost-effective health care practices [18].

### Industry Perspective

Regarding industry, an economic “Do No Harm” safety checklist would favor AI medical technologies that reduce costs and provide increased financial returns while ensuring patient, health care professional, business, and public health well-being. For example, drug research and development is often a long, costly, and complex process. It can take years from the moment a molecular target is identified to when a drug is developed, approved, and marketed; with many candidates often failing during the trial phases. This makes the drug discovery process inefficient with a high financial risk placed on pharmaceutical industries [19].

Nevertheless, AI is now being used to accelerate the process of drug discovery by using deep learning model analysis that reduces the dependency on slower and expensive physical experiments [20]. These models have been effective in predicting the bioactivity and toxicity of potential drugs, identifying medication that fights antibiotic-resistant bacteria in experimental models, and designing a drug that inhibits the discoidin domain receptor 1 (DDR1; a receptor associated with several morbidities, including fibrosis) in experimental models. Regarding this DDR1 inhibiting drug, it was discovered in 21 days and experimentally tested using deep learning models in 46 days [20]. These models can also identify molecules that differ from existing drugs in a clinically meaningful manner and establish novel pathways for developing tools in fighting drug-resistant pathogens as well as other medical conditions [20].

### Government Perspective

From a public health or government perspective, an economic “Do No Harm” safety checklist would favor AI medical technologies that use health resources efficiently while promoting cost-saving and high-value care when compared with the standard used. According to the Centers for Medicare and Medicaid Services, hospitalizations accounted for the largest amount of national health care expenditures in 2017 and 2018 [21].

Models using AI that predict the likelihood of an avoidable hospitalization can target interventions that prevent adverse health outcomes and reduce both individual as well as public health costs. For example, in a study evaluating risk-of-hospitalization (ROH) models using natural language processing and AI developed by Blue Cross Blue Shield of Louisiana (BCBSLA) compared with the standard DxCG (Cotiviti) risk-score algorithms, the Blue Cross logistic regression model had the highest area under the receiving operator characteristics curve (0.862) based on a 10-fold cross-validation result [22]. The Blue Cross AI model’s predictability demonstrates how regional data can be used accurately to identify patients with a high ROH so that health care professionals can intervene earlier and reduce the financial burden placed on patients as well as health care providers over time [22].

### Economic “No-Go” Case

From an economic “Do No Harm” perspective, not all developers of AI medical technologies demonstrate stakeholder safety before launching their products. For example, concerning ROH AI models, an acute hospitalization event is often dependent on access to and use of health care services. However, both of these variables are influenced by racial and socioeconomic disparities [21]. Disparities in access can result in certain populations being underrepresented in the overall target population and data used to predict an outcome of interest. As a result, the model’s output may reflect systematic biases while policies or interventions that use these models may increase the risk of reinforcing and exacerbating existing inequities [21].

In one study, a commercial prediction algorithm widely used to identify and assist patients with complex medical needs (an approach that affects millions of people) in the United States was found to have a significant racial bias. For example, at a given risk score, Black patients were found to be significantly sicker than White patients in most active chronic illnesses [23]. To remedy this disparity, the algorithm should have increased the percentage of Black patients receiving additional care from 17.7% to 46.5%. However, its predictions were based on health care costs rather than illness measures. Unequal access to health care means less money is spent caring for Black patients when compared with White patients and, thus, allows for racial biases to arise when using this approach [23].

This study’s findings highlight the economic risks AI algorithms can have on patients (focusing on cost of care instead of treating or managing the illness), health care professionals (increasing litigation risk due to inadequate care provided), industry (creating distrust and litigation risks from patients as well as health care professionals due to misrepresented patient care metrics), and public health institutions (increasing the cost of care for low socioeconomic and racial minority patients due to worsening health status overtime). It also led to US government officials calling for greater transparency and accountability across the health care industry concerning how AI algorithm use is audited and predictive model biases can be avoided [21].

## Social Factor—Does the AI Medical Technology Cause Social Benefit or Harm in Terms of Access to Affordable Quality of Care to the Stakeholders Involved?

Across medicine, bioethics, public health, and business, there is increasing awareness that novel technology can affect society asymmetrically. Some populations may benefit, others might be harmed, and some may experience no effect at all. These outcomes can cause or worsen existing inequities and exacerbate social factors at all levels of society [24].

Social determinants of health are nonmedical factors that influence health outcomes. They impact health inequities and identify preventable differences in health status between individuals. Several examples of social determinants of health include access to affordable and quality health care services, income and social protection, social support, employment, and education [25]. However, providing access to affordable quality of care is often viewed as a leading objective in improving medical outcomes in a health care system [26]. For this reason, the third factor to consider when deciding if an AI medical technology should be launched is whether its benefits outweigh its harms in providing access to affordable quality of care.

### Patient Perspective

From a patient perspective, a “Do No Harm” safety checklist would favor AI medical technologies that reduce barriers to affordable quality of care while providing improved or equally effective medical services when compared with the standard used. For example, an AI-based electronic interviewer named “Ellie” created by researchers in the United States increased access to care by asking patients questions similar to those made by clinicians [10]. Using a patient’s verbal response, facial expressions, and vocal intonations, Ellie was able to detect signs of depression as well as other medical and psychiatric morbidities that were then followed up by clinicians. In a randomized study, participants were informed that Ellie was controlled by either a human or computer program. Interestingly, participants who were informed that Ellie was controlled by a computer program revealed more personal information [10].

### Health Care Professional Perspective

Deciding whether an AI medical technology is launched should also be based on weighing its social benefits and risks to health care professionals. For instance, as clinical AI systems mature, the quality of health care is expected to improve through reduced human error and physician fatigue or burnout [27]. AI that can perform routine clinical tasks might reduce physician workload and improve the quality of care they provide by allowing for increased time on more demanding responsibilities as well as building rapport with patients. An example of this is an AI medical technology that facilitates triaging and reviewing images so ophthalmologists can spend more time performing surgeries or discussing treatment plans with patients [27].

### Industry Perspective

Regarding industry, a “Do No Harm” safety checklist would favor AI medical tools that improve access to affordable quality of care, ensure stakeholder safety, and promote product exposure as well as use. For example, convolutional neural networks trained on 129,450 clinical images recently achieved dermatologist-level accuracy in diagnosing skin malignancy. This deep learning algorithm also outperformed the average dermatologist in assessing photographic and dermoscopic images. Although its training phase might be expensive, this AI tool can be deployed on mobile devices and potentially improve accessibility of skin lesion screening at the expert level globally [27].

### Government Perspective

From a public health or government perspective, a social “Do No Harm” safety checklist would favor AI medical technologies that increase the scalability of affordable quality of care when compared with the standard used. For example, a histopathological assessment is considered the gold standard for diagnosing multiple types of cancer [27]. However, there are several limitations to this technique, which include pathologist visual evaluation discrepancies, restricted scalability, and underuse of histopathology image features (due to human visual limitations) that help predict cancer survival outcomes [27]. With the development of deep convolutional neural networks, AI can be used to detect prostate cancer from biopsy specimens, breast cancer metastasis in lymph nodes, and mitosis in breast cancer. With an estimated net deficit of over 5700 full-time pathologists in the United States by 2030, an AI system could offset this staff shortage, provide quick and objective histopathology evaluations, and improve accessibility of patients with cancer for quality care [27].

### Social (Access to Affordable Quality of Care) “No-Go” Case

From a social (access to affordable quality of care) “Do No Harm” perspective, not all developers of AI medical technologies demonstrate stakeholder safety before launching their product. For example, a study conducted in Thailand found Google Health’s medical AI-trained tool used to identify signs of DR in patients with diabetes actually reduced quality of care as it was not designed for the clinical environments where it was deployed [28]. Compared with the standard system used in Thailand, nurses taking photos of patients’ eyes during checkups and sending them for analysis by a specialist (a process that can take up to 10 weeks), Google Health’s AI medical tool was able to identify signs of DR from an eye scan with more than 90% accuracy (human specialist level) and provide results in less than 10 minutes. Nevertheless, this finding was based on experimental lab results and not on a real-world setting [28].

After being deployed across 11 clinics spread throughout Thailand, nurses using Google’s AI medical tool were interviewed regarding their experience with it. When working, it facilitated expedited eye scans. However, this AI technology was trained on high-quality scans and rejected images below a certain threshold [28]. With nurses scanning dozens of patients each hour and often taking photos in poor lighting conditions, over 20% of images were excluded. Patients with rejected images were then informed to visit a specialist at another clinic on another day, which was inconvenient if they took time-off work for their appointment or had limited access to transportation. Nurses also expressed frustration when they believed rejected scans showed no signs of disease and follow-up appointments were unnecessary [28]. Another concern was the system had to upload images to the cloud for processing, but limited internet connection in several sites caused delays and hindered the industry and government’s efforts to provide access to quality care in resource-poor settings [28].

## How to Implement the BPES Framework—A Novel Cost-Effective, Time-Sensitive, and Stakeholder Safety-Oriented Quantitative and Qualitative Clinical Phased Trial Approach

Similar to clinical and drug intervention–based studies, randomized controlled trials (RCTs) are considered the gold standard for demonstrating the safety and efficacy of AI-based medical technologies. However, only a limited number of AI RCTs have been published or are registered [29]. Moreover, the findings from these studies are often not generalizable outside the study population and have a limited duration or population size to assess long-term treatment as well as adverse effects. Finally, the increasingly high cost of conducting RCTs creates a dependency on proxy markers that may not correlate well with study outcomes of interest and hinder industry research as well as development due to financial risk [30].

As conducting RCTs can be challenging, other study designs that provide valid evidence for clinical, industry, and public health decision makers should be considered when assessing the safety of launching an AI medical technology. For example, matched cohort, quasi-experimental interrupted time series analyses, prospective pre-and-post, and observational studies can all generate evidence concerning an AI medical technology’s safety and efficacy. Although findings from these study designs might be considered of lower quality when compared with RCTs, they create a compromise between the needs of stakeholders (patients, health care professionals, and public health institutions) seeking timely clinical safety and efficacy knowledge as well as reduce the cost burden for industry [29].

**Figure 2:**
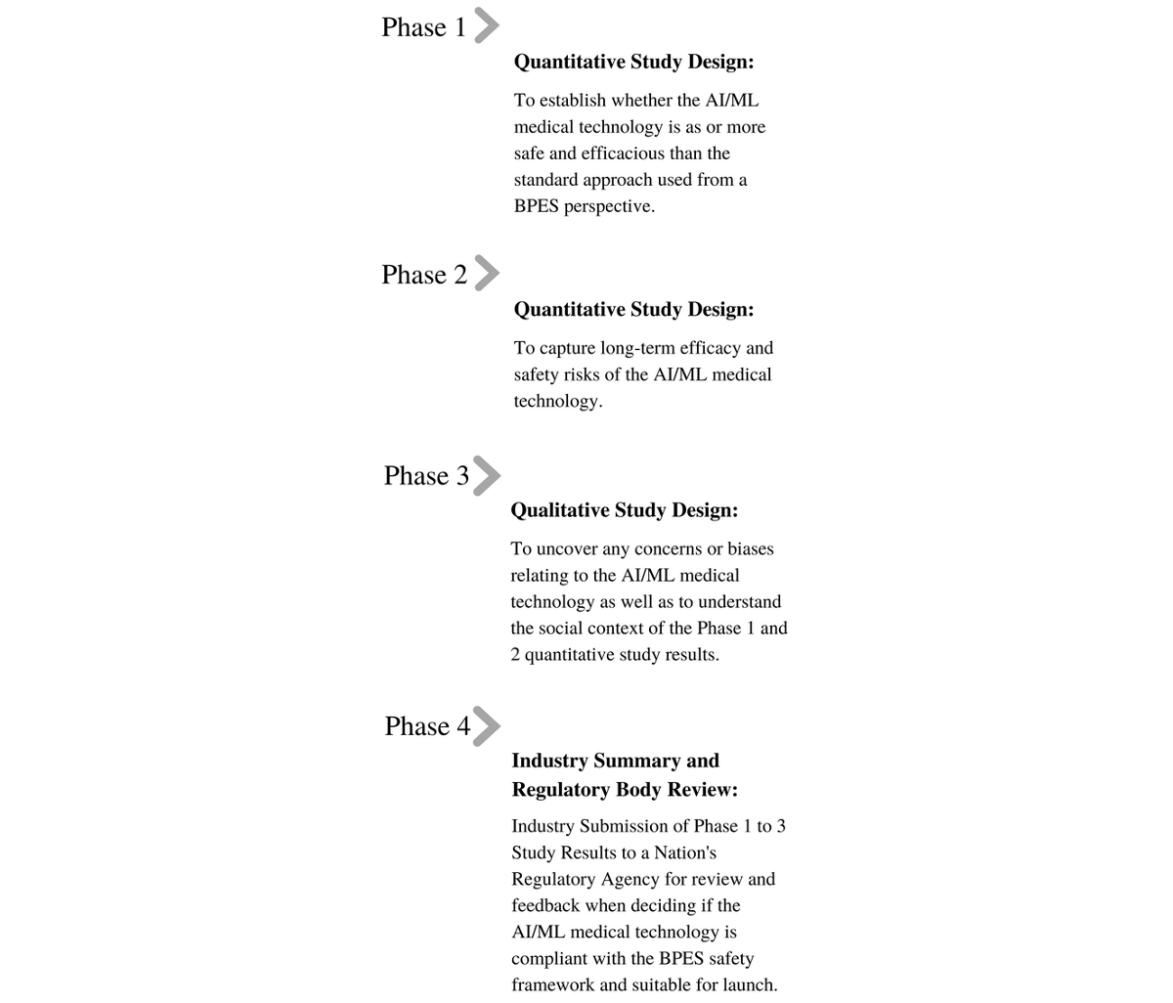
A novel 4-phase study trial approach for assessing whether to launch an AI-based medical technology using the BPES framework. AI: artificial intelligence; BPES: biological-psychological, economic, and social; ML: machine learning.

Beyond quantitative studies to assess the safety and efficacy of AI medical technologies, qualitative research approaches should be used to provide context to the findings observed. For this reason, we recommend a series of studies similar to the phases used in drug trials to determine if an AI medical technology meets the BPES framework’s safety criteria to be launched. This can be achieved using a quantitative study design in the initial phase (phase 1) to establish whether the AI medical technology is as or more safe and efficacious from a BPES perspective than the standard approach used. If phase 1 study findings are positive, a second quantitative study design should be conducted to capture long-term benefits and risks of the AI tool (phase 2). Next, a qualitative study (phase 3) should be performed to uncover any concerns or biases relating to the technology as well as understand the social context of the quantitative study results. Finally, the results of the phase 1 to 3 studies should be summarized and provided to a nation’s health care regulatory agency (ie, Health Canada or the FDA) for review and feedback (phase 4). These phases would ensure a “Do No Harm” BPES safety approach is met and the AI medical technology is safe to launch (Figure 2).

To guarantee, ethical conduct is performed across the study phases, collaborations between industry and patients (via participatory action research study designs and advocacy groups), health care professionals (via academic institutions), and governments (via regulatory agencies) are encouraged. Although using a phase study approach might reduce the speed of launching an AI medical technology, it ensures accountability and transparency and aims to prevent harm to all stakeholders involved.

## Conclusions

The continued development of AI medical technologies (hardware devices, software programs, and mobile apps) is inevitable, but this does not mean there should be no accountability for ensuring they are launched with stakeholder safety in mind. Current frameworks often fail to focus on what makes medicine multidimensional, which is the interactions between patients, health care professionals, industry, and government agencies vis-à-vis BPES factors that can cause and mitigate medical conditions. To fully understand the potential of these AI medical technologies, a framework that places stakeholder safety from a BPES “Do No Harm” perspective is needed. To our knowledge, the BPES framework is the first to do this by placing the Hippocratic Oath of “Do No Harm” at the center of developers, implementers, regulators, and users’ mindsets. Although validation of our framework is needed, its compatibility with existing and future AI reporting guidelines should allow industry AI technology developers, researchers, and government regulatory agencies to explore its efficacy.

## Abbreviations

AI: artificial intelligence
BCBSLA: Blue Cross Blue Shield of Louisiana
BPES: biological-psychological, economic, and social
DDR1: discoidin domain receptor 1
DECIDE-AI: Developmental and Exploratory Clinical Investigations of Decision support systems driven by Artificial Intelligence
DR: diabetic retinopathy
FDA: Food and Drug Administration
MHRA: Medicines and Healthcare Products Regulatory Agency
OECD: Organisation for Economic Co-operation and Development
RCT: randomized controlled trial
ROH: risk-of-hospitalization
